# A dopamine metabolite stabilizes neurotoxic amyloid-β oligomers

**DOI:** 10.1038/s42003-020-01490-3

**Published:** 2021-01-04

**Authors:** Rodrigo Cataldi, Sean Chia, Katarina Pisani, Francesco S. Ruggeri, Catherine K. Xu, Tomas Šneideris, Michele Perni, Sunehera Sarwat, Priyanka Joshi, Janet R. Kumita, Sara Linse, Johnny Habchi, Tuomas P. J. Knowles, Benedetta Mannini, Christopher M. Dobson, Michele Vendruscolo

**Affiliations:** 1 Centre for Misfolding Diseases, Department of Chemistry, University of Cambridge, Cambridge, CB2 1EW UK; 2 Institute of Biotechnology, Life Sciences Center, Vilnius University, Vilnius, 10257 Lithuania; 3 Department of Biochemistry and Structural Biology, Center for Molecular Protein Science, Lund University, Lund, Sweden; 4 Cavendish Laboratory, University of Cambridge, Cambridge, CB3 0HE UK

**Keywords:** Chemical biology, Neuroscience

## Abstract

Aberrant soluble oligomers formed by the amyloid-β peptide (Aβ) are major pathogenic agents in the onset and progression of Alzheimer’s disease. A variety of biomolecules can influence the formation of these oligomers in the brain, although their mechanisms of action are still largely unknown. Here, we studied the effects on Aβ aggregation of DOPAL, a reactive catecholaldehyde intermediate of dopamine metabolism. We found that DOPAL is able to stabilize Aβ oligomeric species, including dimers and trimers, that exert toxic effects on human neuroblastoma cells, in particular increasing cytosolic calcium levels and promoting the generation of reactive oxygen species. These results reveal an interplay between Aβ aggregation and key biochemical processes regulating cellular homeostasis in the brain.

## Abstract (web summary)

Cataldi et al. investigates the impact of the dopamine derivative DOPAL on the Aβ peptide oligomer formation. They report that DOPAL promotes the formation of stable Aβ oligomers that exert toxicity on neuroblastoma cells by increasing cytosolic calcium levels and generating reactive oxygen species. This study connects Aβ aggregation with processes regulating cellular homeostasis in the brain.

## Introduction

Alzheimer’s disease (AD) is an untreatable neurodegenerative disorder that affects over 50 million people worldwide^1–4^. If disease modifying treatments are not found, the number of AD patients is expected to treble by 2050, causing further tremendous human, social and economic impact on our society^1–5^.

The aggregation of Aβ plays a central role in the cascade of events leading to AD, such as loss of synaptic signalling, mitochondria dysfunction, increased production of reactive oxygen species (ROS), neuroinflammation and ultimately, neuronal loss^2,6,7^. At the molecular level, the aggregation process of Aβ starts with the formation of soluble oligomeric species, which further self-assemble into the mature and insoluble fibrils found in the characteristic plaques^1,3,8–10^. Although Aβ plaques are a major hallmark of AD, the severity of the pathology does not correlate well with the extent of amyloid plaque formation^11,12^, but rather with the amount of soluble Aβ oligomers^13–21^, which are thought to be the most toxic species^22–24^. Recent studies have reinforced this significance through the isolation of neurotoxic dimers and other higher-order oligomeric assemblies from the brains of patients^14,25,26^. Because such characterisation remains challenging due to the transient and metastable nature of Aβ oligomers in the brain^23,27^, many studies have attempted to stabilise and characterise non-fibrillar species in order to improve our understanding of oligomeric conformations and toxicity^28–31^. As, however, these aggregates are mostly formed by artificial means, it is important to further investigate their physiological relevance. Moreover, in cellular environments many factors contribute to, and interfere with, the aggregation of Aβ, thereby further increasing the challenges in understanding the nature and interactions of Aβ oligomers^31–34^.

Growing evidence shows that Aβ can be found not only in the extracellular space but also in the luminal part of intracellular compartments and organelles, such as the Golgi complex^35^, endoplasmic reticulum and intermediate compartment^36,37^, and mitochondrial membranes^29,34^. The build-up of intracellular Aβ has been considered an early event in the development of the pathogenesis of AD, and it is also observed in Down syndrome individuals^38–42^. Thus, a multitude of different molecules, both in the intracellular and extracellular spaces, can interact directly with Aβ and affect its aggregation process by modifying its rate of aggregation^43,44^, or redirecting the process towards the formation of off-pathway toxic oligomeric species^31^. Dysregulations in the homoeostasis of these molecules can generate imbalances in their concentrations and activities, in turn altering the aggregation processes^31,32,43,45–47^. Given the complex scenario in which Aβ aggregation takes place, the unravelling of the interactions with other molecules in the crowded intracellular and extracellular spaces, and of the molecular pathways involved, is crucial for a deeper understanding of the molecular origins of AD.

**Fig. 1:**
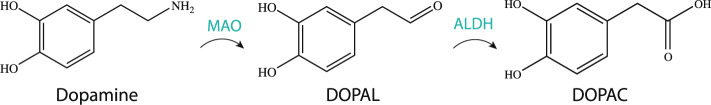
Schematic of the main metabolic pathway involving DOPAL. The catecholamine neurotransmitter dopamine is oxidised into the catecholaldehyde DOPAL (3,4-dihydroxyphenylacetaldehyde) by monoamine oxidases (MAO), and further converted into the less toxic catecholic acid DOPAC (3,4-dihydroxyphenylacetic acid) by aldehyde dehydrogenases (ALDH).

Well-established links exist between neurodegenerative and metabolic disorders, including the observations of direct effects of metabolite dyshomeostasis on synaptic dysfunction and increased oxidative stress^48–53^. Excessive oxidative stress increases the production of toxic and highly reactive aldehydes that are able to interact promiscuously with proteins, resulting in a disruption of protein homoeostasis and cellular damage^52,54^. The monoamine oxidase (MAO) product of dopamine (DA), 3,4-dihydroxyphenylacetaldehyde (DOPAL), is one such aldehyde (Fig. 1), which has previously been shown to induce toxicity in Parkinson’s disease by promoting oligomerisation of α-synuclein and impairing the physiological function of synaptic vesicles (catecholaldehyde hypothesis)^55,56^.

In this study, we show that DOPAL interacts with common isoforms of Aβ (Aβ40 and Aβ42) and inhibits their fibril formation, but not their oligomerisation process. Indeed, DOPAL induces the formation of stable oligomeric species, including dimers and trimers of Aβ40 (Aβ-DO), which possess a certain degree of antiparallel β-sheet conformation. These oligomers exert toxic activity in human neuroblastoma cell cultures, including the induction of both calcium influx and the increase in reactive oxygen species (ROS) production. These stabilised species could resemble some populations of oligomers present in the brain, and could offer a biologically relevant model system to the study Aβ oligomers, which is otherwise challenging in on-pathway oligomeric systems that are transient and more heterogeneous. Overall, our study suggests that DOPAL may act as an intracellular co-neurotoxin in the pathophysiology of AD associated with Aβ oligomers.

## Results

### DOPAL inhibits Aβ40 and Aβ42 fibril formation

**Fig. 2:**
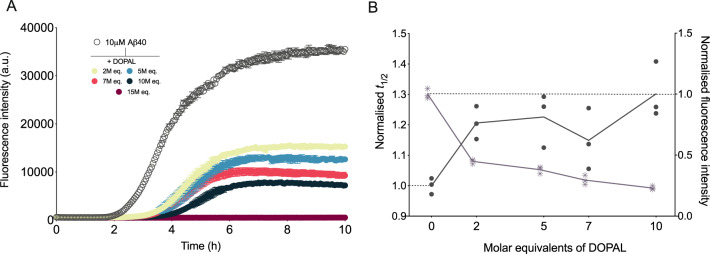
DOPAL inhibits Aβ40 fibril formation in a dose-dependent manner. **a** Aggregation kinetics of Aβ40 in NaP pH 6.9, 0.2 mM EDTA in the absence (empty circles) and presence (filled circles) of increasing molar equivalents of DOPAL (represented in different colours). **b** Normalised to control (absence of DOPAL) half-time (*t*_1/2_) of aggregation (points) and end point fluorescence intensities (asterisks) as derived from the data in **a**. Throughout, error bars represent means ± SEM of three replicates.

DOPAL has been found to interact with a range of proteins, including glucocerebrosidase, ubiquitin and α-synuclein^57^. In particular, it has been observed that DOPAL is able to covalently modify α-synuclein by inducing the formation of α-synuclein oligomers that do not tend to aggregate into cross-β fibrils^54,55^. However, the effects of DOPAL on the aggregation of Aβ are yet to be fully elucidated. To this end, using a highly sensitive chemical kinetics assay based on thioflavin T (ThT) fluorescence, we monitored the aggregation of Aβ40 in the absence and presence of DOPAL at Aβ40-to-DOPAL ratios of 1:2, 1:5, 1:7, 1:10 and 1:15 molar equivalents (Fig. 2). We observed that in the presence of DOPAL, the half-time (*t*_1/2_) of the aggregation was increased as compared to its absence. Furthermore, the final ThT fluorescence intensity, which reflects the concentration of ThT-active fibrils at the end of the reaction, was also decreased in the presence of DOPAL. Both effects were found to be dependent on the concentration of DOPAL (Fig. 2). Notably, in the presence of 2 molar equivalents of DOPAL, we observe a 1.2 fold increase in *t*_1/2_ with a 2-fold reduction in the final fluorescence intensity of the reaction, and in the presence of 15 molar equivalents of DOPAL, the aggregation was arrested entirely and no increase in ThT fluorescence was observed (Fig. 2). Similar effects were also observed on the aggregation of Aβ42, with a decrease in the endpoint ThT fluorescence intensity, as well as an increase in the *t*_1/2_ of aggregation (Fig. S1). Taken together, these results show that DOPAL inhibits fibril formation of both Aβ40 and Aβ42.

**Fig. 3:**
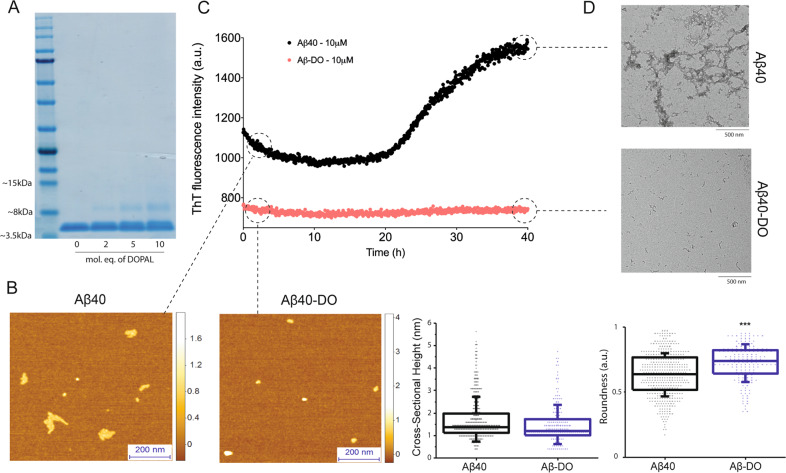
Biophysical characterisation of DOPAL-induced stable Aβ40 oligomers (Aβ-DOs). **a** SDS-page analysis of the pellet fraction of Aβ40 after 20 h of incubation with increasing concentrations of DOPAL. **b**–**d** Morphologies and stability measurement of Aβ40-noDOs and Aβ-DOs. **b** Representative 3D morphology maps measured by AFM of preformed Aβ40-noDOs and Aβ-DOs, with their cross-sectional height and roundness distributions as collected from **a**. **c** ThT-based stability measurements of Aβ40-noDOs and Aβ-DOs over the course of 40 h. **d** TEM images of the samples in **b**, after 40 h of incubation at 37 °C. Note the distinct fibrillised samples of Aβ40-noDOs after the stability measurement, which is otherwise absent in the Aβ40-DOs samples. For figures **b**, **c** and **d**, assays were performed using the sample containing 10 molar equivalents of DOPAL.

Given that the inhibitory effect observed on Aβ fibril formation underlines a molecular interaction between Aβ and DOPAL, we explored whether or not DOPAL can stabilise intermediate Aβ oligomeric species. Specifically, we focused on studying the interaction of DOPAL with the Aβ40 isoform, as it is the most abundantly produced isoform, and its relative concentration with respect to Aβ42 is implicated in the pathology of AD^15,58^. Aβ40 was incubated for 20 h in the absence and in the presence of DOPAL and then subjected to centrifugation. In order to avoid the interference of DMSO in our subsequent analysis, further experiments and characterisation of oligomeric species were performed using the pelleted sample where samples could be resuspended in buffer to remove the traces of the DMSO solvent, albeit similar species were also observed in the supernatant fraction (Figs. 3a and S2). Through an SDS-PAGE analysis, we found that in the absence of DOPAL, 35% of Aβ40 had converted into higher-order pelleted assemblies that could be isolated by centrifugation. In the presence of DOPAL, while approximately a similar 35% of Aβ40 had converted into higher-order pelleted assemblies, we also observed the appearance of a band at a molecular weight slightly above 8 kDa, suggesting the formation of dimeric species in the pellet fraction. The band intensity was observed to increase from about 5% to 15% (of the total intensity of all the bands) with increasing concentrations of DOPAL, indicating a dose-dependent effect on the amount of Aβ species formed at approximately 8 kDa (Fig. 3a).

### DOPAL induces the formation of stable Aβ40 oligomers (Aβ-DOs)

We then investigated the morphologies of these Aβ40 aggregates using phase controlled and high-resolution atomic force microscopy (AFM), which enables characterisation at the single molecule scale and with angstroms resolution of monomers and early oligomeric aggregates. We observed that both in the absence and presence of DOPAL, aggregated samples of Aβ40 possessed similar cross-sectional heights in the order of 1–2 nm (Fig. 3b). The Aβ-DOs have a spherical shape that is typical of oligomeric intermediates^27^, whereas the aggregates formed in the absence of DOPAL showed a tendency to elongate into species that resemble early protofibrils (~1–2 nm cross-sectional height) that would eventually form mature fibrils (~3–6 nm cross-sectional height). A single molecule statistical analysis confirmed that the aggregated species in the presence of DOPAL (Aβ-DOs, *n* = 154) were found to be significantly more spherical (*p* < 0.001) as compared to the species formed in its absence (Aβ-noDOs, *n* = 438) (Fig. 3b). These observations were further substantiated when we assessed the stability of these oligomers over the course of 40 h by ThT kinetics (Fig. 3c). Briefly, after centrifugation, pellets formed in the absence (Aβ40-noDOs) and presence of DOPAL (Aβ-DOs) were diluted to 10 μM, and their stabilities were assessed by means of ThT fluorescence over the course of 40 h at 37 °C. We observed that Aβ-noDOs eventually proceeded to aggregate and convert into fibrils, resulting in an increase in ThT intensity. Conversely, Aβ-DO species showed no change in ThT intensities over time, suggesting a relatively higher stability of these species at 37 °C in comparison to Aβ40-noDOs (Fig. 3b). The transmission electron microscopy (TEM) analysis on these samples obtained after 40 h of the stability measurements confirmed the presence of mature fibrils in the aggregated species of Aβ40 in the absence of DOPAL, and non-fibrillar intermediate species in its presence (Fig. 3d). Thus, these results suggest that unlike Aβ samples which typically aggregate to form fibrillar species, the presence of DOPAL induces the formation of spherical stable Aβ intermediates.

### Aβ-DOs are characterised by an antiparallel β-sheet core and low hydrophobicity

**Fig. 4:**
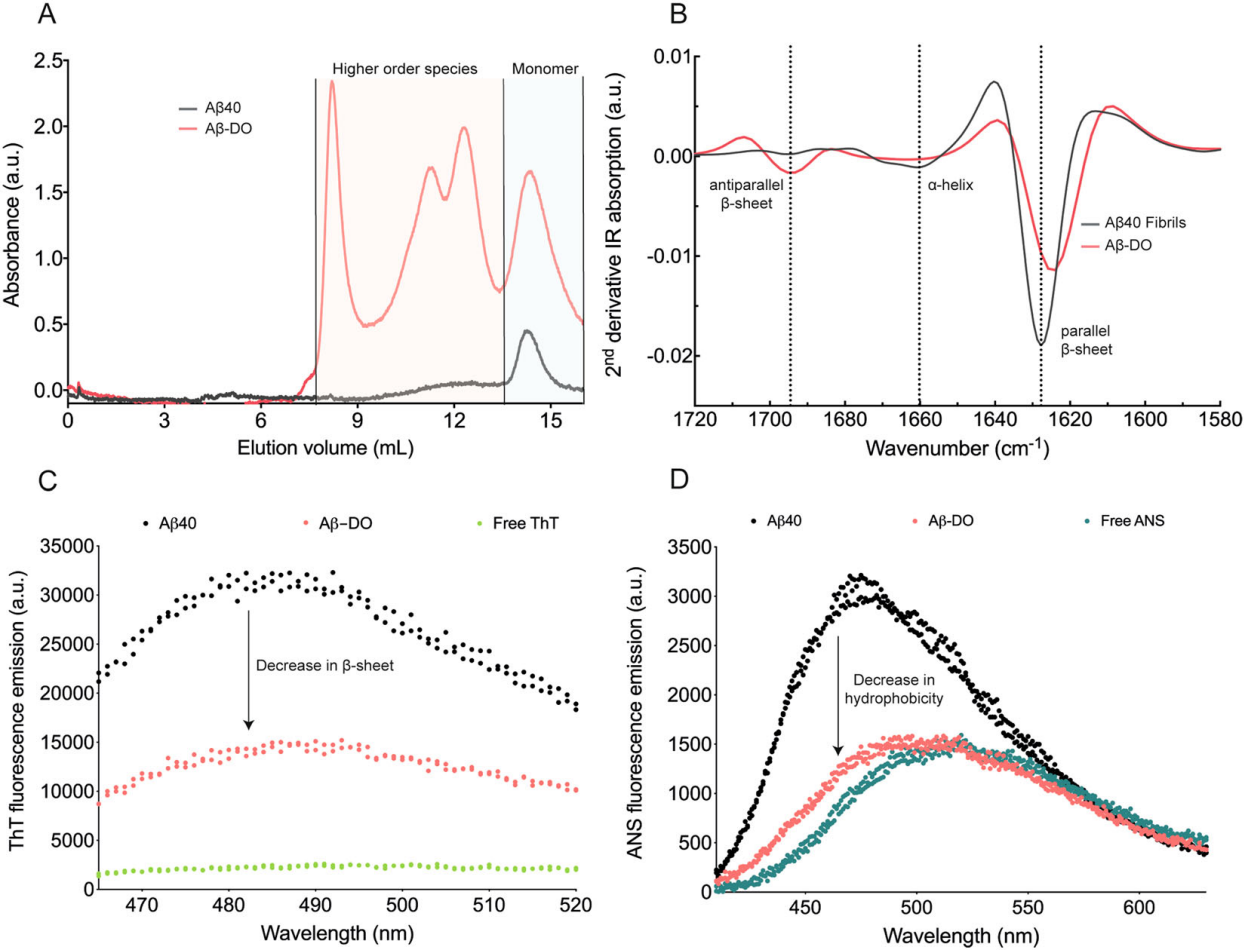
Size distribution and structural characterisation of Aβ-DOs. **a** Size-exclusion chromatography profiles of Aβ40-noDOs (black line) compared to Aβ-DOs (red line). **b** Second derivative of ATR-FTIR spectra of Aβ40 fibrils (black line) and Aβ-DOs (red line). **c** Fluorescence emission spectra of ThT in the absence (green), or in the presence of Aβ40-noDOs (black) or Aβ-DOs (red). **d** Fluorescence emission spectra of ANS in the absence (green), or in the presence of Aβ40-noDOs (black) or Aβ-DOs (red).

In order to further investigate the structural features of the aggregated species formed in the presence of DOPAL and, in particular, those at different molecular weights as observed by SDS-PAGE (Fig. 2a), we performed size exclusion chromatography (SEC) to evaluate the size distribution of the species formed upon reacting with and without DOPAL for comparison. Samples were boiled for 10 min with the addition of 0.1% SDS before injecting it onto the column to assess the distribution profile. We observed in the sample containing Aβ40 with DOPAL, the clear presence of higher-order species, in addition to the monomer fraction. The eluted species between 8 and 13 ml corresponding to the presence of higher-order species were, however, not present in the sample of Aβ40 without DOPAL (Fig. 4a). In spite of the same volume and concentration (monomer equivalents) injected into the column, we note the diverse qualitative nature of the two SEC profiles: this difference might be due to aggregated samples stuck at the pre-filter of the column in the sample Aβ40-noDO, and the more heterogenous nature of the samples Aβ40-DO responsible of the broad absorbance peaks. SEC profiles allow us to assess the presence of higher-order species for Aβ-DO, but more sophisticated techniques such as sedimentation velocity profiles are warranted to accurately quantify such ensembles. As a further control, matrix-assisted laser desorption/ionisation (MALDI) mass spectrometry analysis of both samples without the addition of 0.1% SDS confirmed that while a dominant signal corresponding to 4462 Da (Aβ(M1-40) monomer) was present in both Aβ40-noDO and Aβ-DO samples, in the case of Aβ-DO, other signals corresponding to higher-order species of dimers (8920, 8936 Da) and trimers (13398 Da) could also be observed, which is otherwise much less significant with respect to the Aβ40-noDO sample (Figs. S3 and S4). These results also suggest that higher-order Aβ species formed in the presence of DOPAL as observed in SDS-PAGE and SEC were not artefactually induced by the presence of SDS. We also observed from the molecular weight of the dimers and trimers that the species formed are composed of Aβ alone and not in complex with DOPAL, suggesting that oligomerisation may be driven by non-covalent interactions with the metabolite (Fig. S3).

We then investigated the secondary structure of Aβ-DOs using attenuated total reflection Fourier transform infrared (ATR-FTIR) spectroscopy (Fig. 4b). Aβ-DOs showed a strong signal at 1625 cm^−1^, which correlates with a substantial presence of intermolecular parallel β-sheet structure, and a strong signal at 1693 cm^−1^, which indicates the presence of antiparallel β-sheet structure^59^. The antiparallel β-sheet structure has been reported as a characteristic of both Aβ40 and Aβ42 oligomeric species^60–62^. This was further confirmed by comparing the FTIR spectrum of Aβ-DOs with fully formed Aβ40 fibrils, where we only observed a parallel β-sheet peak at 1627 cm^−1^. Thus, the secondary structures of Aβ-DOs and Aβ40 fibrils are different. In addition, we also validated the content of solvent-exposed β-sheet structure of Aβ-noDOs via a ThT assay (Fig. 4c). After 20 h of incubation, we observed that Aβ40 samples in the absence of DOPAL exhibited a higher ThT fluorescence signal (15-fold increase) as compared to Aβ40 samples in the presence of DOPAL (5-fold increase), thus indicating a lower content of solvent-exposed β-sheet content in the latter sample. This observation could also be attributed to the higher amounts of fibrillar content in Aβ-noDOs as compared to the oligomeric samples of Aβ-DOs. We also sought to assess the hydrophobicity of the Aβ40 samples, which is considered as a key determinant feature of oligomers^27^. This assessment was performed by measuring the fluorescence change in the dye 8-anilinonaphthalene-1-sulfonic-acid (ANS). Upon binding to hydrophobic surfaces, ANS generates an increase in fluorescence and a blue-shift of its maximum emission peak wavelength^63^. While Aβ-noDOs showed an approximately 2-fold increase in the fluorescence intensity when compared to the control and a blue-shift of its maximum wavelength, Aβ-DOs caused only a slight blue-shift and a small increase in fluorescence emission (Fig. 4d). Taken together, these results show that Aβ-DOs are oligomeric with a core β-sheet structure, with low content of β-sheet and hydrophobic patches exposed to the solvent.

### Aβ-DOs are toxic to human neuroblastoma cells

Aβ oligomers are known to cause toxicity to cells, including by enhancing the production of ROS, disrupting Ca^2+^ influx and promoting mitochondrial dysfunction^31,64,65^. Thus, we evaluated the ability of Aβ-DOs to cause cellular dysfunction to human SH-SY5Y cells using three different assays, the 3-(4,5-dimethylthiazol-2-yl)-2,5-diphenyltetrazolium bromide (MTT) reduction test, the measurement of Ca^2+^ influx and the detection of ROS production.

**Fig. 5:**
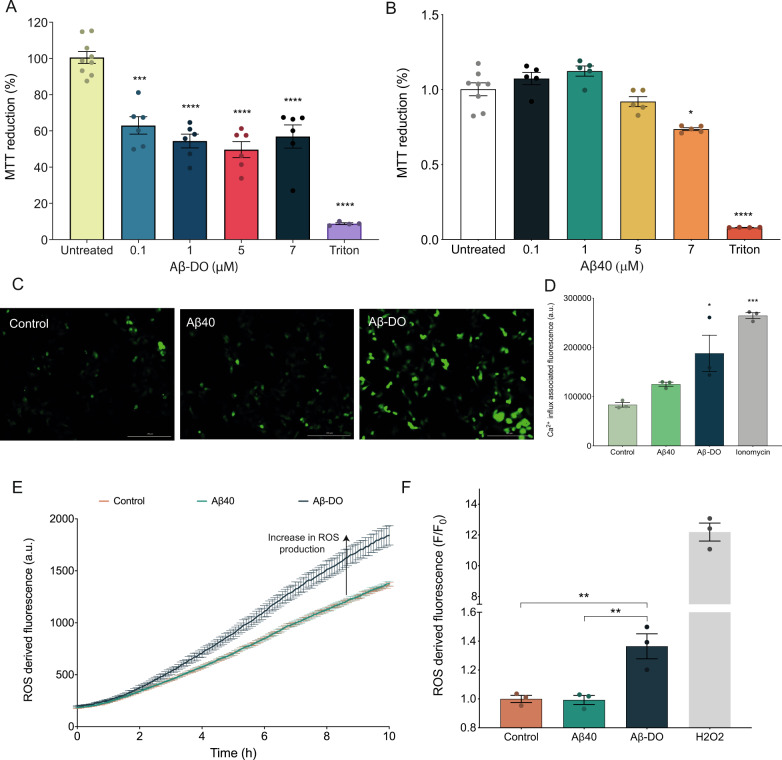
Aβ-DOs are toxic to neuroblastoma cells. **a** Cell viability determined by the MTT reduction test in cells exposed to increasing concentrations of Aβ-DOs. **b** Intracellular Ca^2+^ influx associated fluorescence upon 40 min of treatment with buffer (light green), Aβ40-noDOs (green) or Aβ-DOs (dark green). F/F_0_ is the ratio between the Ca^2+^ influx-derived fluorescence intensity in the presence (F) and absence (F0) of Aβ aggregates. **c** Representative images of intracellular Ca^2+^ influx upon 40 min treatment with buffer (left), Aβ40-noDOs (centre) or Aβ-DOs (right). **d** Kinetics of ROS production of cells at 37 °C either in the absence or in the presence of Aβ40-noDOs or Aβ-DOs. **e** F/F_0_ ratio between the ROS-derived fluorescence intensity in the presence (F) and absence (F_0_) of Aβ40-noDOs (green) and Aβ-DOs (dark green) at 10 h; the untreated control is shown in orange. Positive control of 0.01% H2O2 is shown in grey. For the MTT test, error bars represent means ± SEM of five replicates. For calcium influx and ROS production, error bars represent means ± SEM of three replicates. *p ≤ 0.05, **p ≤ 0.01, ***p ≤ 0.001, ****p ≤ 0.0001 relative to untreated cells are shown.

Firstly, we evaluated viability of the SH-SY5Y cells after 24 h of treatment with Aβ incubated in the presence or in the absence of DOPAL. The MTT test indicated that the cellular dysfunction following the treatment with Aβ-DOs increased. The viability of the cells decreased in 33 ± 6% in the presence of 0.1 μM Aβ-DO and reached the highest decrement of 47 ± 5% at 5 μM. The treatment of the cells with 7 µM Aβ-DO was found to cause a significantly different toxic effect compared to the untreated, but not to increase further this signal, in line with previously published data showing that the toxicity of the oligomers correlates non-linearly with their concentration^66^ (Fig. 5a). Since DOPAL alone did not induce significant toxicity, the toxicity observed could thus be attributed to the presence of Aβ-DOs (Figs. 5a, S6 and S7). In contrast, although Aβ-noDOs showed toxicity, the decrease in cell viability was only significant at the highest concentration tested (Fig. 5b). To get insight into the biological events occurring at the early stages after the treatment, we evaluated the Ca^2+^ influx and the ROS production in cells upon exposure to Aβ40-noDOs and Aβ-DOs. The cells treated with Aβ-DOs showed a significant increase in fluorescence after 40 min of treatment, indicating Ca^2+^ influx into the cells, whereas cells treated with Aβ40-noDOs did not show a significant increase (Fig. 5c, d). Following the addition of Aβ-DOs to the cells a significant increase in fluorescence was observed over time, reflecting an increased production of ROS triggered by Aβ-DOs (Fig. 5e, f). Conversely, cells exposed to Aβ40-noDOs did not display a significant ROS production (Fig. 5e, f). Overall, we could observe significant toxicity to neuroblastoma cells caused by the exposure to Aβ-DOs, which manifests in the reduction of cell metabolic activity (MTT test), increase in Ca^2+^ influx, and increase in ROS production. We note that these effects are comparable to previous reports, where oligomeric species of Aβ and α-synuclein were shown to cause an increase in ROS production, as well as cellular toxicity, though it is possible that other toxicity mechanisms may also account for the overall cytotoxic activity of the oligomers, including unspecific interactions with membrane receptors, such as NMDA and AMPA receptors^16,17,24,31^.

## Discussion

Oligomeric species of Aβ are closely implicated in the pathophysiology of AD^13–17^ and have been found in brain extracts^18,19^ and cerebrospinal fluid of AD patients^20^. Recently, cross-linked Aβ dimers found in brain tissue of AD patients were shown to be toxic towards induced pluripotent stem cell (iPSC)-derived neurons^26^. Furthermore, Aβ dimers were found to directly induce hyperphosphorylation of tau^22^, and to impair learning and synaptic plasticity in mice, even in the absence of amyloid plaques^21^, suggesting that such species may play a critical role in the early stages of the disease.

Although the presence of Aβ dimers and other higher order species has been reported by several groups, and their causative role in the pathophysiology of AD is well accepted^13,14,16–21^, the physiological mechanism of their formation still remains unclear. Here, we have observed that DOPAL, an endogenous brain metabolite, leads to the formation of stable neurotoxic oligomers of Aβ40, unveiling possible interactions between metabolites and proteins within the crowded and complex cellular environment. In fact, since physiological concentrations of DOPAL are in excess with respect to that of Aβ, such a mechanism of higher stoichiometry of DOPAL-to-Aβ can also manifest in vivo^9,67–69^.

**Fig. 6:**
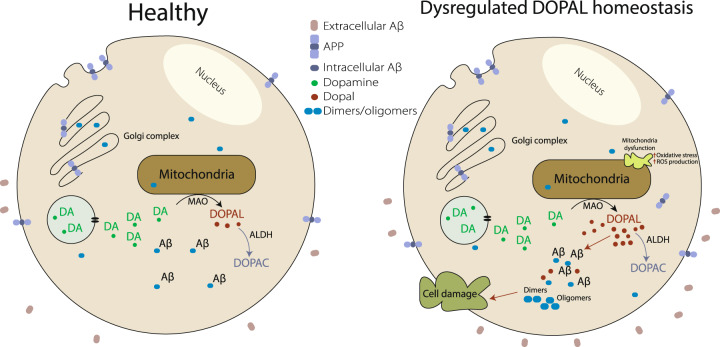
DOPAL dysregulation hypothesis of Aβ oligomerisation. A dysregulation in DOPAL homeostasis and a consequent build-up of DOPAL can lead to the stabilisation of toxic intracellular oligomers of Aβ. This phenomenon leads to cellular damage and further oxidative stress.

Substantial evidence correlates a rise in levels of DOPAL with different pathological processes, such as mitochondrial dysfunction and oxidative stress^52^. It has been shown that aberrant oxidative stress can lead to a build-up of DOPAL, by catalysing dopamine oxidation and blocking the further catabolism of DOPAL into less toxic metabolites^6^. Furthermore, mitochondrial dysfunction and oxidative stress can be induced by several neurological diseases including AD, depression and anxiety disorders^70–74^. Hence, we propose a molecular mechanism where an abnormal build-up of DOPAL can lead to the stabilisation of toxic dimers and other oligomers of Aβ, initiating an early cascade of events that may contribute to the pathophysiology of AD (Fig. 6). Indeed, while homeostatic control allows for the presence of biological factors such as molecular chaperones to inhibit the aggregation of Aβ as a means of reducing toxicity, homoeostatic dysregulation can lead to the build-up of other metabolites, such as DOPAL, which can further trigger the toxicity cascade in the overall pathology of the disease. In this view, this mechanism illustrates how the inhibition of the fibril formation could lead to deleterious effects if associated to the stabilisation of intermediate species, and how reactive metabolic intermediates can influence early events in the development of AD. Understanding these mechanisms can lead to new approaches in prevention and treatment of AD and other protein aggregation diseases.

## Methods

### Production of Aβ40 and Aβ42 peptides

Recombinant Aβ(M1-40) (MDAEFRHDSGYEVHHQKLVFFAEDVGSNKGAIIGLMVGGVV), here called Aβ40, and Aβ (M1-42) (MDAEFRHDSGYEVHHQKLVFFAEDVGSNKGAIIGLMVGGVVIA), here called Aβ42, were expressed and purified using a method previously described^10^. In particular, Aβ(M1-40) has been shown to aggregate and adopt a similar fibril structure as Aβ(1-40)^75^. Briefly, *Escherichia coli* BL21-Gold (DE3) (Stratagene, La Jolla, CA) expressing the peptides were sonicated and inclusion bodies were dissolved in 8 M urea, followed by anion exchange in batch mode using diethylaminoethyl cellulose resin and lyophilisation. Combined lyophilised fractions were further purified using a Superdex 75HR 26/60 column (GE Healthcare, Chicago, IL) and eluates were analysed using SDS–polyacrylamide gel electrophoresis NuPAGE 4–12% Bis-Tris for the presence of the desired protein product. The fractions containing the pure peptide were lyophilised again and stored in −80 °C.

### Preparation of Aβ40 and Aβ42 for kinetic experiments

Solutions of Aβ40 and Aβ42 were prepared by dissolving the lyophilised Aβ40 or Aβ42 in 6 M guanidinium hydrochloride (GnHCl). Monomers were separated from salt and potential fibrillar or oligomeric species using a Superdex 75 Increase 10/300 GL column (GE Healthcare) at a flow rate of 0.5 mL/min, eluted in 20 mM sodium phosphate buffer, pH 6.9 (Aβ40) and pH 8 (Aβ42), supplemented with 200 μM EDTA. The peak corresponding to the monomer fraction was collected and the concentration of the peptide was determined by the absorption of the integrated peak area using ε280 = 1490 l mol^−1^ cm^−1^. The resulting monomer solution was diluted with the respective buffer used in the purification to the desired concentrations and supplemented with 20 μM ThT. Samples were then incubated in the absence or presence of different molar equivalents of DOPAL (3,4-dihydroxyphenylacetaldehyde) (ChemCruz, Dallas, TX), which were prepared from a stock concentration of 200 mM, and then pipetted into multiple wells of a 96-well half-area, low-binding, clear bottom and PEG coated plate (Corning 3881), 80 µL per well.

### Preparation of Aβ40 oligomers

Aβ40 oligomers were prepared as described previously^31^. To generate the Aβ40 oligomers, 0.5 mg of lyophilised peptide was dissolved in 300 μL hexafluoro-2-isopropanol (HFIP), incubated overnight at 4 °C, and dried under a gentle N_2_ flow to evaporate the HFIP. The peptide was resuspended in 50 μL DMSO, sonicated for 10 min at room temperature, diluted in sodium phosphate buffer, at pH 6.9, in the absence or presence of different concentrations of DOPAL to a final concentration of 100 μM, and incubated at 20 °C for 20 h in a final volume of 275 μL. After the reaction, the oligomeric fractions were isolated via centrifugation at 15,000 rpm for 20 min at 20 °C, and resuspended in 275 μL to a final concentration of 35 μM (as determined from SDS-PAGE).

### Aβ40 oligomers stability assay

After 20 h of incubation, samples containing Aβ40 (10 μM monomer equivalent) in the absence and presence of 10 molar equivalents of DOPAL were added to a 20 μM ThT solution and further incubated in a plate reader at 37 °C for 40 h. The fluorescence intensity was recorded using an excitation 440 nm and emission 480 nm wavelenghts with a plate reader ClarioStar (BMG Labtech, Aylesbury, UK).

### Atomic force microscopy (AFM)

After 20 h of incubation, samples containing Aβ40 (35 μM monomer equivalents) in the absence or presence of 10 molar equivalents of DOPAL were diluted to 0.5 μM (monomer equivalents), then deposited on freshly cleaved mica substrates using AFM. Ten microlitre diluted samples were deposited on the substrate at room temperature. The samples were incubated for 10 min, followed by rinsing with 1 mL milliQ water. Then the samples were dried using a gentle flow of nitrogen gas. AFM maps of 3D morphology of all the samples were acquired in the regime of constant phase change, with 2 nm/pixel resolution using a NX10 (Park Systems, city, South Korea) operating in non-contact mode^76^. This set-up was equipped with a silicon tip with a nominal radius of <8 nm and spring constant of 5 N/m (PPP-NCHR).

Scanning Probe Image Processor (SPIP) (Image Metrology, Denmark) software was used for image flattening and single aggregate statistical analysis. The average level of noise for each image is in the order of <0.1 nm, as demonstrated by roughness measurements^77,78^. All the measurements were performed at room temperature.

### Transmission electron microscopy (TEM)

After the oligomer stability measurement, samples containing Aβ40 in the absence and presence of 10 molar equivalents of DOPAL were diluted to 2 μM (monomer equivalents), and prepared on 400-mesh, 3-mm copper grid carbon support film grids (EM Resolutions Ltd., Sheffield, UK) and stained with 2% (w/v) uranyl acetate solution. Salts and excess uranyl acetate were washed by rinsing with deionized water (Milli-Q) and dried at RT over 10 min. Imaging was carried out using the FEI Tecnai G2 transmission electron microscope (Cambridge Advanced Imaging Centre (CAIC) University of Cambridge, UK), and the images were acquired using the SIS Megaview II Image Capture system (Olympus, Muenster, Germany).

### Size exclusion chromatography

Separation of different Aβ40 species in the presence or absence of 10 molar equivalents of DOPAL after 20 h of incubation was obtained by gel filtration using a Superdex^TM^ 75 10/300 GL column (GE Healthcare) connected to an ÄKTAprime (GE Healthcare) system. The column was equilibrated with phosphate buffer pH 6.9 and the elution was followed at a flow rate of 0.5 ml/min. The absorbance was measured at 280 nm.

### Fourier transform infrared spectroscopy (FTIR)

Oligomeric samples of Aβ40 stabilised with 10 molar equivalents of DOPAL, as well as fibrillar samples of Aβ40 were centrifuged and the pellets resuspended in 12 μL of phosphate buffer at pH 6.9 to a concentration of 2.6 mM (monomer equivalent of protein). Attenuated total reflection FTIR (ATR-FTIR) spectroscopy was performed using a Bruker Vertex 70 spectrometer with a diamond ATR element (Bruker, Billerica, MA). The spectra were acquired with a resolution of 4 cm^−1^ and processed using the Bruker software. Four spectra were averaged (each spectrum obtained from 128 scans), and the second derivative was calculated using a Savitzky−Golay filter (2nd order, 12 points).

### ThT endpoint binding assay

After 20 h of incubation, samples containing Aβ40 (10 μM monomer equivalent) in the absence and presence of 10 molar equivalents of DOPAL were added to solutions of ThT in phosphate buffer at pH 6.9 to a final concentration of 20 μM ThT. The emission spectra of excitation for ThT (440 nm) were acquired at 25 °C using the plate reader ClarioStar (BMG Labtech, Aylesbury, UK). Samples were analysed from two independent preparations and results show an average of 40 scans for each sample.

### ANS binding assay

After 20 h of incubation, samples containing Aβ40 (10 μM monomer equivalent) in the absence or presence of 10 molar equivalents of DOPAL were added to solutions of 8-anilinonaphthalene-1-sulfonic-acid (ANS) in phosphate buffer at pH 6.9 to obtain 3-fold excess of ANS. The emission spectra of excitation for ANS (380 nm) were acquired at 25 °C using the plate reader ClarioStar Omega (BMG Labtech, Aylesbury, UK). Samples were analysed from two independent preparations and results show an average of 40 scans for each sample.

### Cell cultures

Human SH-SY5Y neuroblastoma cells (A.T.C.C., Manassas, VA) were cultured in Dulbecco’s Modified Eagles Medium (DMEM)/F12+GlutaMax supplement (Thermo Fisher Scientific, Waltham, MA) with 10% heat-inactivated foetal bovine serum. The cell cultures were maintained at 37 °C in a 5.0% CO_2_ humidified atmosphere and grown until 80% confluence for a maximum of 20 passages.

### MTT reduction assay

Cell viability was measured using the 3-(4,5-dimethylthiazol-2-yl)-2,5-diphenyltetrazolium bromide (MTT) reduction assay. Cells were transferred to into a 96-well plate and treated for 24 h with samples containing Aβ40 in the absence or presence of 10 molar equivalents of DOPAL after a 20 h incubation. The cells cultures were then incubated with 0.5 mg/mL MTT solution at 37 °C for 4 h, followed by the addition of cell lysis buffer (20% SDS, 50% N,N-dimethylformamide, pH 4.7) for 3 h. Absorbance values of blue formazan were measured at 590 nm using a plate reader (ClarioStar Omega BMG Labtech, Aylesbury, UK), and cell viability was expressed as the percentage of MTT reduction in treated cells as compared to untreated cells (taken as 100%).

### Ca^2+^ influx assay

Cytosolic calcium ions (Ca^2+^) levels were measured by exposing the SH-SY5Y cells loaded with 2.0 μM Fluo4-AM (Thermo Fisher Scientific, Waltham, MA) to samples containing Aβ40 in the absence or presence of 10 molar equivalents of DOPAL after a 20 h incubation. The emitted fluorescence of Fluo4 was recorded after excitation at 488 nm using the fluorescence microscope Cytation5 Cell Imaging Reader and quantified by means of the Gen5 Data Analysis software (BioTek Instruments, Winooski, VT).

### Measurement of ROS production

ROS production was measured using the Fluorometric Intracellular ROS kit MAK143 (Sigma-Aldrich, St. Louis, MO) following the manufacturer’s protocol. Briefly, SH-SY5Y cells were seeded in black polystyrene 96-well plates for 24 h, followed by treatment with 1 μM of samples containing Aβ40 in the absence or presence of 10 molar equivalents of DOPAL after a 20 h incubation. The ROS production was monitored over time exploiting the fact that ROS species react with a fluorogenic sensor in the cytoplasm, resulting in a fluorometric product whose amount is proportional to the amount of ROS present. We thus measured the emission of fluorescence at 520 nm (excitation at 490 nm) at 37 °C using a plate reader (BMG Labtech, Aylesbury, UK).

### Statistics and reproducibility

For cellular assays, comparisons between groups were performed using the one-way ANOVA followed by Bonferroni’s multiple comparison test. A *p*-value lower than 0.05 was considered statistically significant. *p < 0.05, **p < 0.01, ***p < 0.001 and ****p < 0.0001.

### Reporting summary

Further information on research design is available in the Nature Research Reporting Summary linked to this article.

## Supplementary information

Supplementary Information

Description of Additional Supplementary Items

Supplementary Data 1

Reporting Summary

## Data Availability

The data generated or analysed in this study are included in the article and supporting information. All data are available from the authors upon reasonable request. All data generated or analysed during this study are included in this published article (and its supplementary information files).
