# Publisher Correction: A dopamine metabolite stabilizes neurotoxic amyloid-β oligomers

**DOI:** 10.1038/s42003-021-01680-7

**Published:** 2021-01-26

**Authors:** Rodrigo Cataldi, Sean Chia, Katarina Pisani, Francesco S. Ruggeri, Catherine K. Xu, Tomas Šneideris, Michele Perni, Sunehera Sarwat, Priyanka Joshi, Janet R. Kumita, Sara Linse, Johnny Habchi, Tuomas P. J. Knowles, Benedetta Mannini, Christopher M. Dobson, Michele Vendruscolo

**Affiliations:** 1 Centre for Misfolding Diseases, Department of Chemistry, University of Cambridge, Cambridge, CB2 1EW UK; 2 Institute of Biotechnology, Life Sciences Center, Vilnius University, Vilnius, 10257 Lithuania; 3 Department of Biochemistry and Structural Biology, Center for Molecular Protein Science, Lund University, Lund, Sweden; 4 Cavendish Laboratory, University of Cambridge, Cambridge, CB3 0HE UK

**Keywords:** Chemical biology, Neuroscience

Correction to: *Communications Biology* 10.1038/s42003-020-01490-3, published online 4 January 2021.

In the original published Article there was an error in the labelling of the X axis for Fig. 5B, D and F. In all three cases A! should have appeared as Aβ as correctly denoted in panel A.

