# Supplementary material for: A dopamine metabolite stabilizes neurotoxic amyloid-β oligomers
Source: Commun Biol. 2021 Jan 4;4:19. doi: 10.1038/s42003-020-01490-3 (PMC7782527; doi:10.1038/s42003-020-01490-3)
Supplement: Supplementary file 1 — Supplementary Information [file 42003_2020_1490_MOESM1_ESM.pdf]

Supporting information

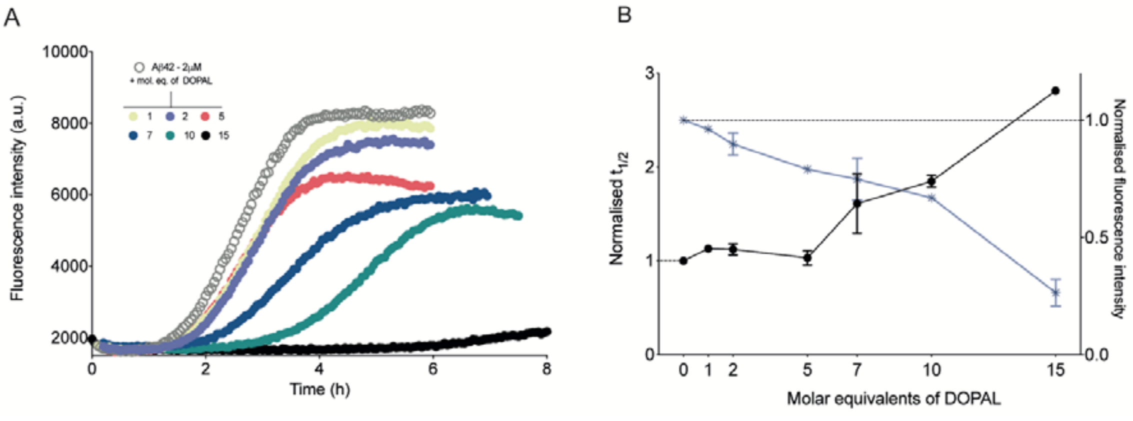

Figure S1 | DOPAL inhibits Aβ42 fibril formation in a dose-dependent manner. (A) Aggregation kinetics of Aβ42 in NaP, pH 8.0, 0.2 mM EDTA in the absence (empty circles) and presence (filled circles) of increasing molecular equivalents of DOPAL (represented in different colours). **(B)** Normalised half-time ($t_{1/2}$) of aggregation (points) and normalised end point fluorescence intensities (bars) as derived from the data in (A). Throughout, error bars represent means ± SEM of three replicates.

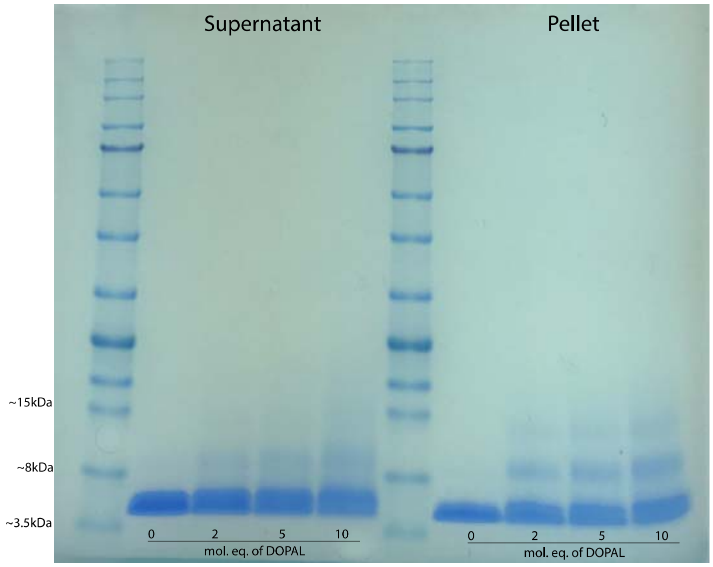

Figure S2 | DOPAL induces the formation of stable high ordered species of Aβ40. (A) SDS-page analysis of the supernatant and pellet fraction of Aβ40 after 20 h of incubation with increasing concentrations of DOPAL.

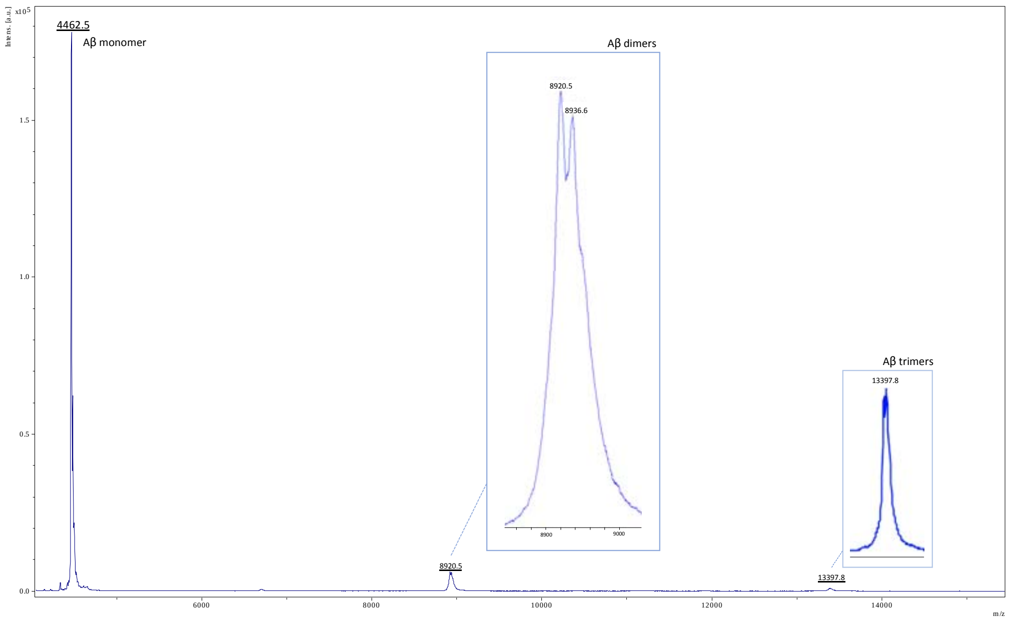

Figure S3 | MALDI of Aβ-DOs. MALDI mass spectra of Aβ-DOs. Aβ-DOs contain various other species besides the monomers (4462 Da), such as dimers (8920, 8936 Da), trimers (13398Da) and other higher molecular weight species.

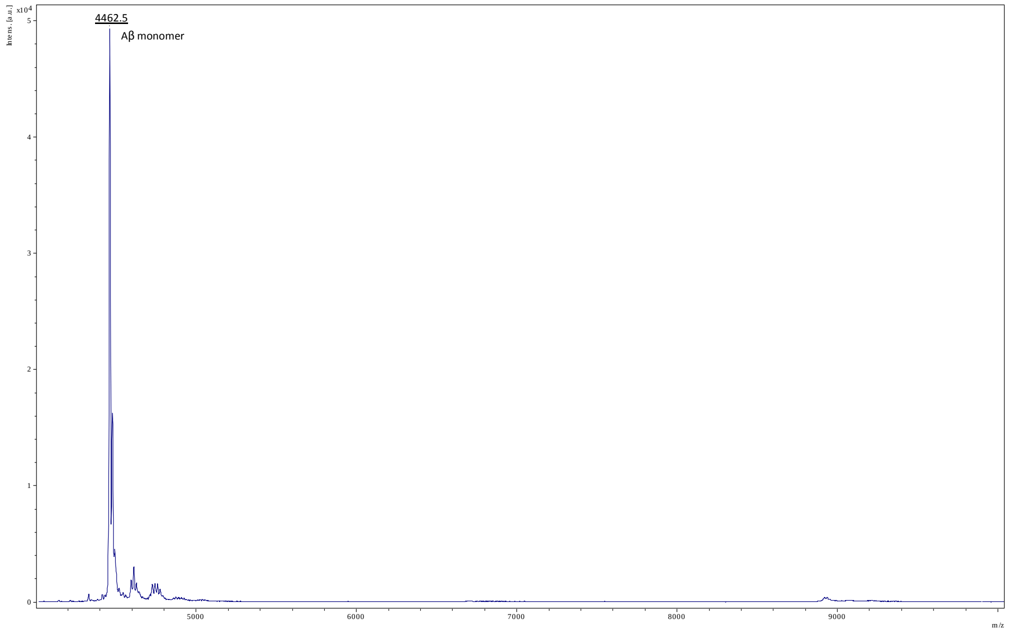

Figure S4 | MALDI of Aβ40-noDO samples. MALDI mass spectra of Aβ40-noDO samples, which were incubated in the absence of DOPAL for 20 h at 20 °C. Aβ40-noDO aggregates are not stable and dissociate in this assay, leaving only the Aβ40 monomer peak at 4462.5 Da. The additional low peaks up to 5000 Da can be attributed to oxidated forms of Aβ40 monomers.

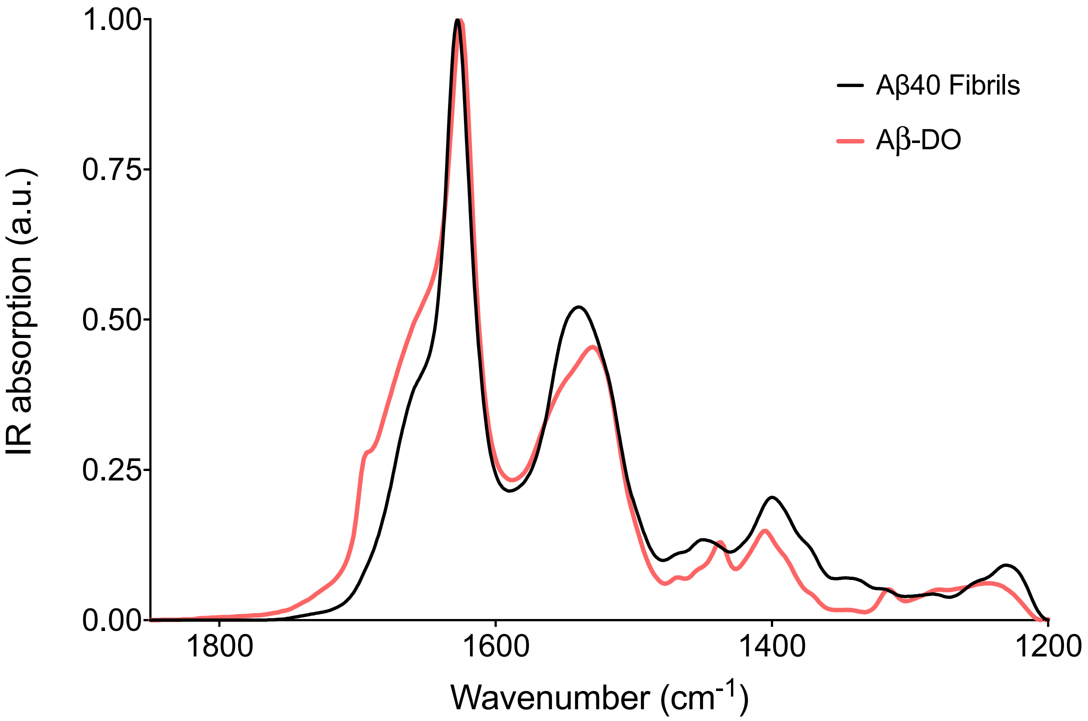

Figure S5 | ATR-FTIR spectra of Aβ40 fibrils and Aβ-DOs.

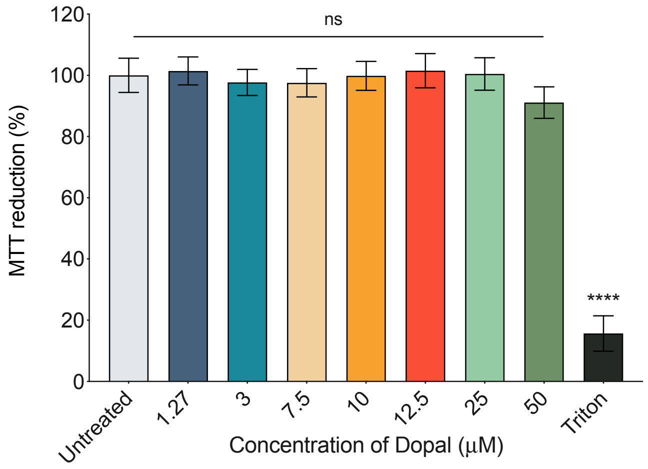

Figure S6 | Cytotoxicity of DOPAL in human neuroblastoma SH-SY5Y cell cultures. Cytotoxicity of DOPAL at a range of different concentrations measured by means of MTT reduction and compared to untreated control. Throughout, error bars represent means ± SEM of six replicates from two independent experiments. The change in cell viabilities of samples measured from (A) and (B) (except in the presence of Triton) were all found to be not significant as compared to the untreated sample.

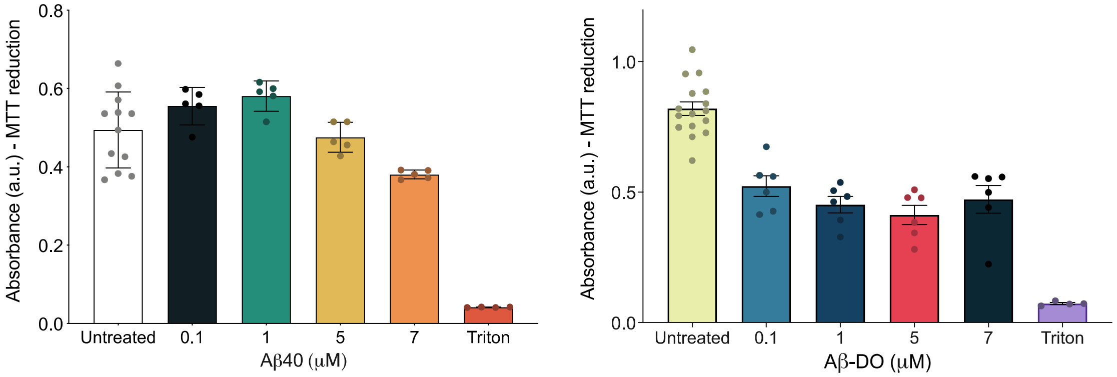

Figure S7 | Cytotoxicity of Aβ40 and Aβ-DO in human neuroblastoma SH-SY5Y cell cultures. Raw absorption values corresponding to MTT reduction of Aβ40 (Aβ40-noDos) and Aβ-DO.
